# Influence of Different Organic Waste Materials on the Transformation of Nitrogen in Soils

**DOI:** 10.1100/tsw.2001.390

**Published:** 2001-12-08

**Authors:** D.K. Das, A.M. Puste

**Affiliations:** Department of Agricultural Chemistry and Soil Science, Bidhan Chandra Krishi Viswavidyalaya, Nadia, West Bengal, India

**Keywords:** ammoniacal nitrogen, hydrolysable nitrogen, nitrate nitrogen, nonhydrolysable nitrogen, organic waste materials, submerged soil, transformation

## Abstract

Organic waste materials like crop residues, well-decomposed cow dung, composts, and other rural and urban wastes are considered highly useful resources in enhancing soil fertility and also in build-up of soil organic matter. Organic matter decomposition provides plant nutrients in soil, which in turn increases crop productivity. Availability of nutrients and nitrogen (N) and phosphorus from organic waste materials is dependent upon the nature of organic residues, climatic conditions, and soil moisture activity. Keeping these factors in view, the present investigation was undertaken to study the transformation of N from different organic waste materials in two contrasting soils from an eastern India, subtropical region. The results showed that the amounts of ammoniacal-N (NH_4_-N), nitrate-N (NO_3_-N), hydrolysable N (HL-N), and nonhydrolysable (NHL-N) were increased for up to 60 days of soil submergence and increased further with the increase (1% by weight of soil) of organic residue application. Considering the effect of various organic waste materials, it was found that the amounts of NH_4_-N, NO_3_-N, HL-N, and NHL-N were higher with the application of groundnut hull as compared to wheat straw and potato skin, which may be due to relatively narrow carbon:N ratio of groundnut (22:43) than that of wheat straw (62:84) and potato skin (71:32); however, the results showed that the release of NH_4_-N, NO_3_-N, HL-N, and NHL-N was in the order of groundnut hull > wheat straw > potato skin.

## INTRODUCTION

Different types of organic waste materials, like crop residues or rural and urban wastes, are useful in augmenting soil fertility and increasing crop productivity. Most of the Indian subtropical soils are deficient in organic carbon (C) as well as nitrogen (N). Application of these organic waste materials in soils, therefore, helps the building up of plant nutrient status, especially N; however, such build-up of soil fertility status depends upon the nature of waste materials added and the dynamics of mineralisation immobilisation of nutrients (N, phosphorus [P], and sulphur[S]) in soils being mediated by soil microorganisms[1,2,3].

The status of organic N fractions in soils can be determined by hydrolysing the soil with 6N HCl[4]. Bremner[5] reported that more than 95% of the total N was in organic combination, a portion of which, on hydrolysis, is liberated as ammonium (10 to 25%), a greater portion as amino acids (25 to 40%), and a smaller fraction (1 to 5%) as amino sugars. These hydrolysable N (HL-N) fractions are the main source of the availability of N for the nutrition of organisms in the soil[6]. Dhillon et al.[7] reported that the total N, HL-N, and nonhydrolysable N (NHL-N) correlated significantly with the yield and N uptake by rice. Considering some of these factors, the present study was undertaken with an objective to investigate the influence of different organic materials on the transformation of N in soils of the Indian subtropics.

## MATERIALS AND METHODS

The surface (0- to 15-cm depth) soil samples from Mohitnagar (Inceptisol) and Bolpur (Alfisol) in the districts of Jalpaiguri and Birbhum, respectively, were collected. Then soils were crushed and sieved through a 20-mesh sieve. The relevant physicochemical properties of soils *viz.* pH, EC, organic C, cation exchange capacity (CEC), and available N (Table 1) were determined following the method described by Jackson[8]. In the present investigation, potato skin, groundnut hull, and wheat straw were used as organic wastes, and these organic wastes were dried, ground, and analysed for total C and N (Table 2). The levels of all three organic waste materials used at 0.5 and 1.0% by weight of the soil. The experiment was conducted in a laboratory at room temperature (25 ± 2°C) with seven treatments and three replications in a randomised block design. The seven treatments were $T_1$ = control (no application of organic waste materials); $T_2$ and $T_3$ = application of potato skin at 0.5 and 1.0% by weight of the soil, respectively; $T_4$ and $T_5$ = application of wheat straw at 0.5 and 1.0% by weight of the soil, respectively; and $T_6$ and $T_7$ = application of groundnut hull at 0.5 and 1.0% by weight of the soil, respectively. All the soils receiving the above seven treatments were kept submerged in standing water 5 + 0.5 cm above the soil surface, simulating lowland rice conditions. Soils from all three replications were analysed at 15-, 30-, 45-, and 60-day intervals after the treatment for different fractions of N.

## RESULTS AND DISCUSSION

### Ammoniacal Nitrogen ($NH_4$-N)

The results (Table 3) show that the amount of $NH_4$-N increased with the period of submergence; however, the magnitude of such increase varied with the type of soil as well as applied organic waste materials. Comparing the result of different organic waste materials in both the soils, it was observed that the amount released with respect to $NH_4$-N was highest in the $T_6$ and $T_7$ treatments, ranging between 60.2 and 88.1 mg kg$^{-1}$ at 60 days of submergence. These amounts of release of $NH_4$-N in groundnut were twice as high as was observed in potato skin and wheat straw treatments. The order of $NH_4$-N release from these materials was groundnut hull > wheat straw > potato skin. The lower rate of $NH_4$-N release from potato skin and wheat straw treatments can be attributed to narrower C:N ratios of these additives (Table 2), which limits mineralisation of N[9]. The percent increase of $NH_4$-N in both the soils over the control progressively increased up to 60 days of submergence in all organic waste materials and was highest in the case of alfisol treated with 1.0% groundnut hull.

### Nitrate-Nitrogen ($NO_3$-N) Content

The results (Table 4) show that the amount of $NO_3$-N content in both soils gradually increased mineralisation of organic waste

**TABLE 1**
**Physicochemical Properties of the Soil Used for the Experiment**

| Physicochemical Properties | Inceptisol (Mohitnagar Soil) | Alfisol (Bolpur Soil) |
|---|---|---|
| pH | 6.02 | 5.70 |
| EC (dsm$^{-1}$) | 0.17 | 0.49 |
| Organic C (%) | 0.92 | 0.58 |
| CEC [Cmol(p$^+$)kg$^{-1}$] | 23.70 | 7.86 |
| Available $NO_3$-N (mg kg$^{-1}$) | 1.52 | 0.87 |
| Available $NH_4$-N (mg kg$^{-1}$) | 8.34 | 7.50 |
| Available P (mg kg$^{-1}$) | 10.38 | 8.20 |

**TABLE 2**
**Results of Chemical Analysis of the Organic Residues Used for the Experiment**

| Chemical Properties | Potato Skin | Wheat Straw | Groundnut Hull |
|---|---|---|---|
| Total C (%) | 48.50 | 38.96 | 35.45 |
| Total P (%) | 0.38 | 0.60 | 0.44 |
| Total N (%) | 0.68 | 0.62 | 1.58 |
| C:N ratio | 71.32 | 62.84 | 22.43 |
| C:P ratio | 127.63 | 64.93 | 80.56 |

**TABLE 3**
**Effect of Potato Skin, Wheat Straw, and Groundnut Hull on the Availability of $NH_4$-N (mg kg$^{-1}$ soil) in Inceptisol and Alfisol**

| Treatments | 15 | % Increase Over Control | 30 | % Increase Over Control | 45 | % Increase Over Control | 60 | % Increase Over Control | Mean |
|---|---|---|---|---|---|---|---|---|---|
| **Inceptisol (Mohitnagar Soil)** | | | | | | | | | |
| $T_1$ | 9.2 | — | 10.5 | — | 12.5 | — | 14.6 | — | 11.7 |
| $T_2$ | 11.8 | 26.0 | 14.0 | 35.7 | 15.4 | 28.7 | 17.5 | 28.9 | 14.7 |
| $T_3$ | 14.8 | 55.8 | 17.5 | 70.0 | 19.5 | 69.9 | 20.7 | 61.0 | 18.1 |
| $T_4$ | 14.1 | 48.7 | 16.8 | 63.0 | 18.9 | 64.4 | 20.3 | 56.8 | 17.5 |
| $T_5$ | 19.4 | 102.0 | 25.8 | 153.4 | 27.4 | 148.5 | 29.6 | 147.5 | 25.5 |
| $T_6$ | 42.1 | 328.3 | 48.2 | 377.4 | 52.4 | 399.4 | 60.2 | 456.2 | 50.7 |
| $T_7$ | 55.2 | 45.9 | 65.7 | 52.0 | 72.8 | 602.9 | 78.4 | 647.5 | 68.0 |
| **Mean** | 23.8 | | 28.4 | | 31.3 | | 34.5 | | |
| **C.D. (5%)** | 0.2 | | 0.2 | | 1.3 | | 0.8 | | |
| **Alfisol (Bolpur)** | | | | | | | | | |
| $T_1$ | 8.1 | — | 9.2 | — | 11.2 | — | 12.6 | — | 10.3 |
| $T_2$ | 8.7 | 6.3 | 9.8 | 6.5 | 12.3 | 10.3 | 14.0 | 14.8 | 11.2 |
| $T_3$ | 9.8 | 17.1 | 11.5 | 20.5 | 13.9 | 27.0 | 16.7 | 41.7 | 12.9 |
| $T_4$ | 12.5 | 44.5 | 16.2 | 68.7 | 18.1 | 68.7 | 21.6 | 90.2 | 17.1 |
| $T_5$ | 18.8 | 107.0 | 29.9 | 207.2 | 32.1 | 208.3 | 34.1 | 215.7 | 28.7 |
| $T_6$ | 43.4 | 353.0 | 51.7 | 425.7 | 68.3 | 571.0 | 83.2 | 706.0 | 61.6 |
| $T_7$ | 50.7 | 426.4 | 57.3 | 481.2 | 73.4 | 621.7 | 88.1 | 755.7 | 67.4 |
| **Mean** | 21.7 | | 26.5 | | 32.8 | | 38.6 | | |
| **C.D. (5%)** | 0.3 | | 0.2 | | 0.2 | | 0.8 | | |

Period of Submergence (days): 15, 30, 45, 60.

materials with time. The increase in the $NO_3$-N value was much lower as compared to the increase in the amount of $NH_4$-N in both soils. The results further revealed that the release of $NO_3$-N content in both soils showed a similar pattern to that of $NH_4$-N content. A slow increase in $NO_3$-N content in soils with the period of flooding might be the result of constant release of inorganic N from the slow mineralisation of organic resource materials as well as relatively greater thickness of the oxidised soil layer. As a result, it is possible that a higher rate of biological oxidation of $NH_4$-N results in a greater $NO_3$-N content in the oxidised soil layer with the simultaneous decrease in diffusion of $NO_3$-N to the anaerobic, reduced soil layer. Although $NH_4$-N is stable in the reduced zone of the soil, basal application of organic waste materials is usually placed deep enough to avoid immediate nitrification; however, diffusion of $NH_4$-N from the reduced zone to the oxidised zone

$$\left(NH_4 - N \xrightarrow{\text{nitrification}} NO_3 - N\right)$$

cannot be prevented[10]. Beri et al.[11] observed that the use of amendment in the soil with *Sesbania aculeata* resulted in the formation of $NO_3$-N under both field-capacity and saturated-soil conditions. In the case of both inceptisol and alfisol, with up to 60 days of submergence with the application of groundnut hull, wheat straw, and potato skin at 1.0%, the percent increase of $NO_3$-N over control was 174.2, 83.9, and 28.2 (inceptisol), and 118.8, 55.7, and 35.0 (alfisol), for each application, respectively.

## HL-N and NHL-N

The results (Table 5) revealed that the amount of HL-N and NHL-N initially increased significantly; thereafter, the amounts decreased with the progress of submergence irrespective of treatments. The magnitude of such changes, however, varied with treatments and was highest in the treatment with groundnut hull at its highest level (1.0% by weight of soil). This result may be because the organic N of groundnut hull has a relatively low C:N

**TABLE 4**
**Effect of Potato Skin, Wheat Straw, and Groundnut Hull on the Availability of $NO_3$-N (mg $kg^{-1}$ soil) in Inceptisol and Alfisol**

| Treatments | 15 | % Increase Over Control | 30 | % Increase Over Control | 45 | % Increase Over Control | 60 | % Increase Over Control | Mean |
|---|---|---|---|---|---|---|---|---|---|
| **Inceptisol (Mohitnagar Soil)** | | | | | | | | | |
| $T_1$ | 2.1 | — | 2.7 | — | 3.5 | — | 4.2 | — | 3.1 |
| $T_2$ | 2.6 | 5.4 | 3.2 | 4.5 | 4.2 | 7.7 | 5.5 | 13.2 | 3.9 |
| $T_3$ | 3.4 | 13.9 | 4.2 | 14.3 | 5.8 | 23.2 | 7.0 | 28.2 | 5.1 |
| $T_4$ | 3.6 | 15.2 | 4.2 | 14.8 | 6.3 | 27.8 | 8.7 | 45.0 | 5.1 |
| $T_5$ | 7.7 | 56.2 | 11.1 | 83.8 | 11.9 | 84.7 | 12.6 | 83.9 | 5.7 |
| $T_6$ | 11.9 | 98.5 | 14.7 | 119.5 | 17.4 | 139.5 | 19.6 | 154.0 | 10.8 |
| $T_7$ | 13.7 | 116.0 | 16.7 | 139.6 | 19.6 | 161.3 | 21.6 | 174.2 | 18.0 |
| **Mean** | 6.4 | | 8.1 | | 9.8 | | 11.3 | | |
| **C.D. (5%)** | 0.3 | | 0.1 | | 0.2 | | 0.2 | | |
| **Alfisol (Bolpur)** | | | | | | | | | |
| $T_1$ | 1.4 | — | 2.2 | — | 3.4 | — | 4.8 | — | 3.0 |
| $T_2$ | 1.7 | 2.5 | 2.5 | 2.7 | 4.2 | 8.8 | 6.3 | 14.7 | 3.7 |
| $T_3$ | 2.4 | 9.6 | 3.2 | 9.6 | 5.4 | 20.6 | 8.3 | 35.0 | 4.8 |
| $T_4$ | 3.5 | 20.2 | 4.2 | 20.2 | 6.2 | 28.1 | 7.6 | 58.0 | 5.4 |
| $T_5$ | 4.8 | 33.7 | 6.3 | 40.8 | 9.0 | 56.5 | 10.4 | 55.7 | 7.6 |
| $T_6$ | 5.8 | 43.8 | 7.1 | 49.5 | 11.1 | 77.5 | 13.3 | 8.00 | 9.3 |
| $T_7$ | 9.8 | 83.3 | 13.3 | 110.8 | 15.3 | 109.6 | 16.7 | 118.8 | 13.8 |
| **Mean** | 4.2 | | 5.5 | | 7.8 | | 9.6 | | |
| **C.D. (5%)** | 0.2 | | 0.2 | | 0.2 | | 0.3 | | |

Period of Submergence (days): 15, 30, 45, 60.

ratio and is converted to HL-N very easily as compared to other sources of organic materials[12]. Tusneem and Patrick[13] reported that the amount of total HL-N and NHL-N was found to be increased following the application of N-fertiliser. The N from organic sources is converted to HL-N because of microbial decomposition, which varies with the kind of C sources added to the soil. The progressive decline in HL-N content at the later period of submergence may be because of its continuous hydrolysis to amino acids and their subsequent mineralisation to ammonium and nitrate forms[14]. The results further indicate that the magnitude of decrease in the amount of NHL-N was relatively low compared to the HL-N fraction, which may be because of immobilised of mineralised N to nonhydrolysable organic nitrogenfraction[15].

A comparison of the results of different organic treatments revealed that the amounts of both HL-N and NHL-N in both the soils were higher in groundnut hull treatments, closely followed by wheat straw, possibly because of variation in the kinds of organic materials as well as the C:N ratio determining the process of mineralisation of organic resource materials.

## CONCLUSION

From the present investigation it may be concluded that organic agricultural waste materials are very beneficial not only for maintaining soil fertility—particularly in respect to augmenting N content in soils—but also a source of C, which may improve soil physical conditions.

**TABLE 5**
**Influence of Organic Waste Materials on the Changes of HL-N and NHL-N (mg kg$^{-1}$ soil) in Inceptisol and Alfisol**

| | Inceptisol (Mohitnagar Soil) | | | | | | | |
|---|---|---|---|---|---|---|---|---|
| | Period of Submergence (days) | | | | | | | |
| | 15 | | 30 | | 45 | | 60 | |
| Treatments | HL-N | NHL-N | HL-N | NHL-N | HL-N | NHL-N | HL-N | NHL-N |
| $T_1$ | 553.2 | 186.5 | 508.4 | 169.7 | 485.5 | 137.2 | 441.4 | 100.83 |
| $T_2$ | 643.1 | 211.4 | 630.3 | 192.9 | 589.8 | 152.3 | 503.6 | 117.8 |
| $T_3$ | 667.4 | 242.6 | 644.4 | 208.8 | 602.4 | 167.5 | 561.3 | 141.1 |
| $T_4$ | 725.4 | 221.5 | 698.9 | 198.8 | 621.2 | 156.5 | 579.8 | 130.0 |
| $T_5$ | 785.7 | 278.4 | 718.6 | 225.9 | 669.8 | 176.8 | 604.9 | 140.70 |
| $T_6$ | 807.2 | 268.6 | 714.9 | 215.7 | 658.8 | 171.4 | 617.9 | 134.7 |
| $T_7$ | 825.7 | 284.7 | 779.9 | 245.7 | 694.9 | 203.4 | 642.7 | 151.9 |
| C.D. (5%) | 204.7 | 18.4 | 27.7 | 16.9 | 22.4 | 13.8 | 17.3 | 12.9 |
| | Alfisol (Bolpur) | | | | | | | |
| $T_1$ | 481.9 | 138.7 | 403.3 | 118.6 | 387.4 | 100.4 | 300.9 | 89.7 |
| $T_2$ | 502.4 | 149.1 | 431.9 | 130.7 | 412.4 | 106.8 | 381.7 | 94.7 |
| $T_3$ | 529.0 | 158.4 | 502.7 | 139.9 | 473.4 | 119.41 | 404.5 | 101.7 |
| $T_4$ | 539.4 | 154.5 | 509.2 | 141.4 | 419.8 | 110.2 | 389.4 | 99.1 |
| $T_5$ | 585.4 | 171.9 | 532.5 | 158.4 | 488.5 | 126.8 | 428.9 | 114.6 |
| $T_6$ | 558.7 | 161.7 | 515.7 | 149.3 | 451.7 | 113.5 | 417.4 | 103.4 |
| $T_7$ | 591.7 | 185.7 | 536.8 | 164.7 | 498.4 | 144.7 | 442.6 | 131.7 |
| C.D. (5%) | 12.7 | 9.8 | 9.3 | 10.1 | 16.7 | 8.3 | 12.7 | 9.9 |

| | | |
|---|---|---|
| Received: | July | 28, 2001 |
| Revised: | November | 10, 2001 |
| Accepted: | November | 12, 2001 |
| Published: | December | 8, 2001 |

## BIOSKETCH

**Dr. Dilip Kumar Das** is Professor, Department of Agricultural Chemistry & Soil Science, Bidhan Chandra Krishi Viswavidyalaya. He holds B.Sc.(Ag) Hons, M.Sc(Ag), and Ph.D. degrees. Professor Das has made his contribution to the field of soil science, primarily in soil chemistry, soil fertility, and plant nutrition with particular reference to nitrogen and micronutrients including some heavy metal pollution problems in Indian sub-tropics. He has evaluated various channels of nitrogen losses in lowland rice soils vis-à-vis suggested methods for the minimization of those losses (ammonia volatilization, leaching, and denitrification), research that has been included in *Advances in Soil Science*. In addition, Prof. Das has done exhaustive research on mirconutrients in soils and crops. He has published two important books on soil science, namely, *Introductory Soil Science* (1996) and *Micronutrients: Their Behaviour in Soils and Plants* (2000) (both published by Kalyani Publishers, India). These books are useful for students engaged in agricultural education in India and abroad. Prof. Das received the Crop Research Award in 1997, conferred by the Agricultural Research Information Centre, Hisar, India, for his significant contribution in the field of research on various crops and promoting agricultural literature. He was also awarded the 20th Century Agricultural Development National Award in 1999, conferred by the Gaurav Society of Agricultural Research Information Centre, Hisar, India, for his significant contribution in the field of research on soils and crops.
